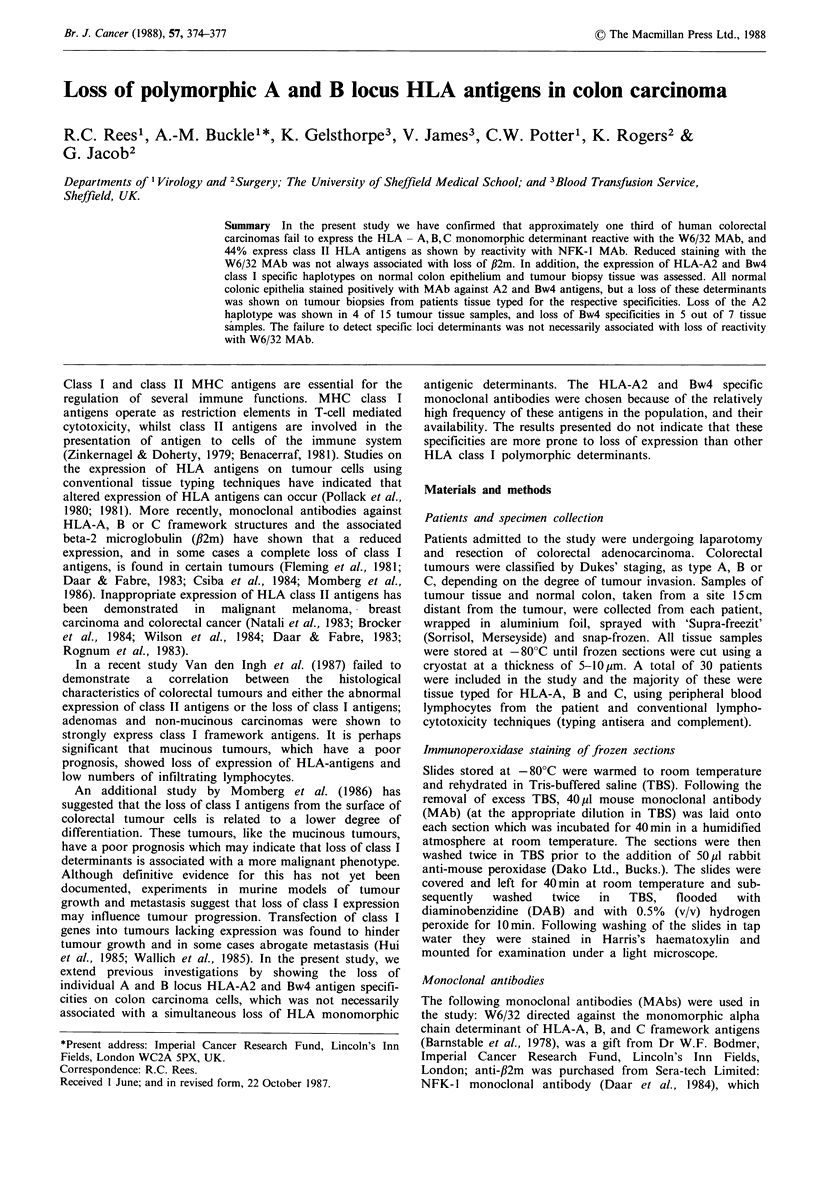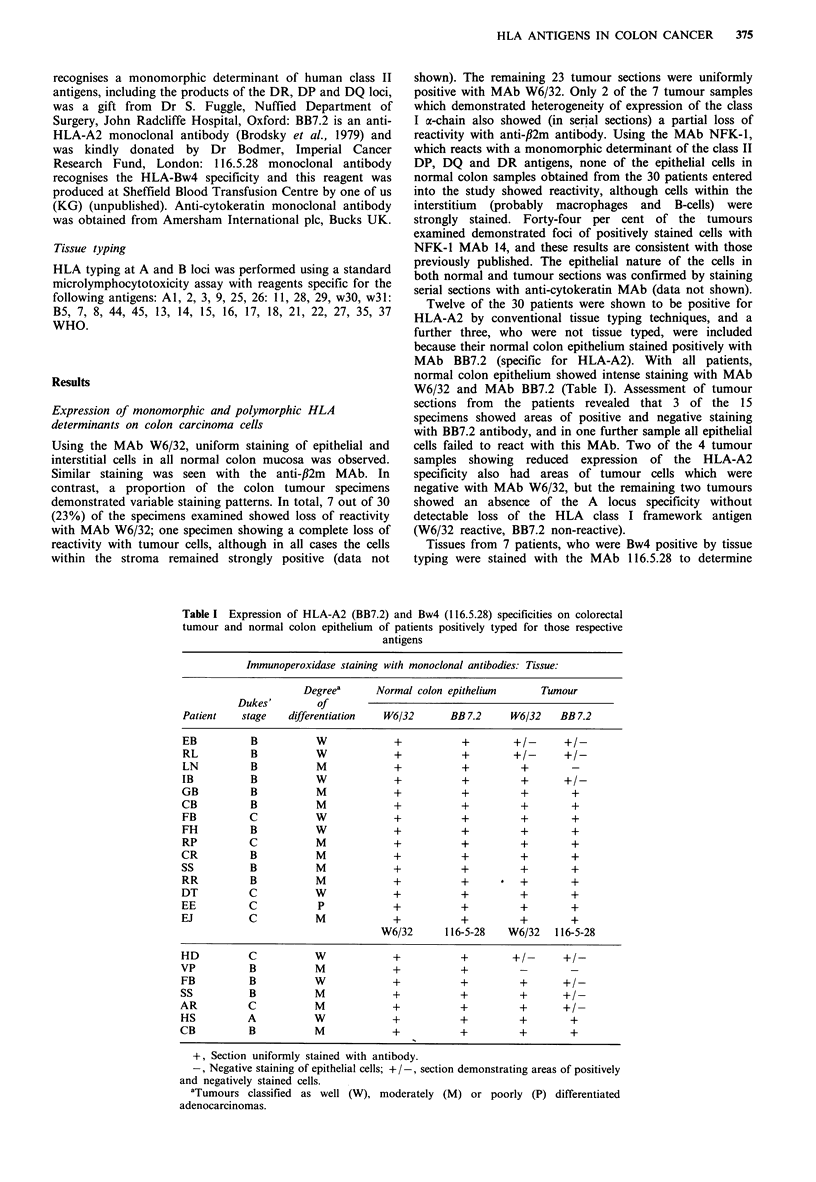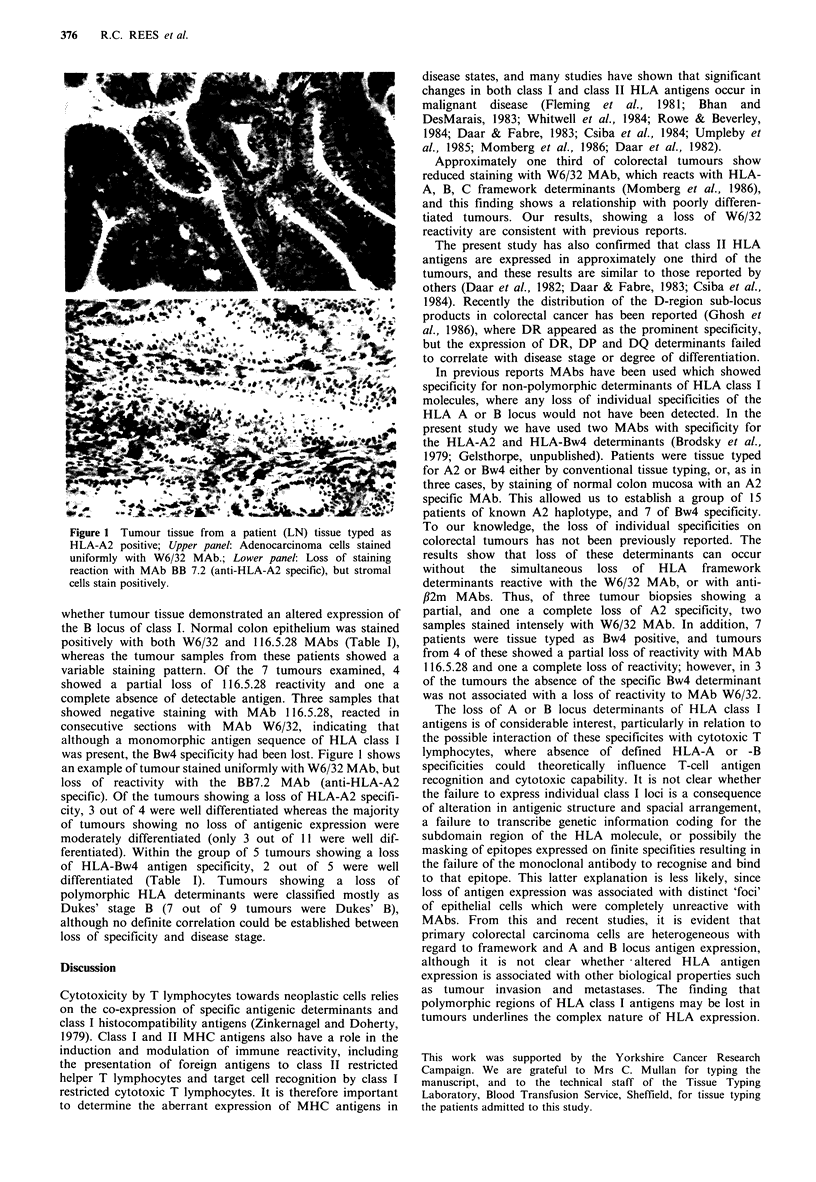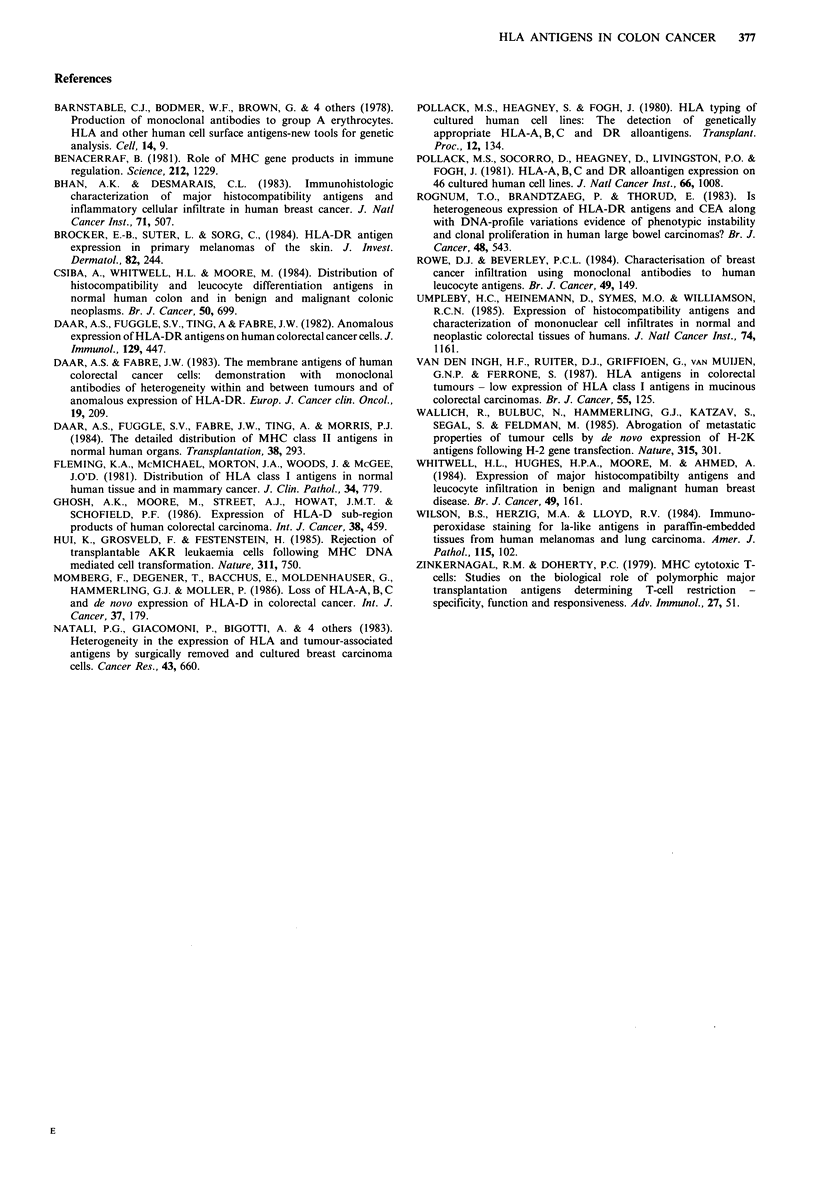# Loss of polymorphic A and B locus HLA antigens in colon carcinoma.

**DOI:** 10.1038/bjc.1988.85

**Published:** 1988-04

**Authors:** R. C. Rees, A. M. Buckle, K. Gelsthorpe, V. James, C. W. Potter, K. Rogers, G. Jacob

**Affiliations:** Department of Virology, University of Sheffield Medical School, UK.

## Abstract

**Images:**


Br. J. Cancer (1988), 57, 374-377 © The Macmillan Press Ltd., 1988

### Loss of polymorphic A and B locus HLA antigens in colon carcinoma

R.C. Rees[1], A.-M. Buckle[1]*, K. Gelsthorpe[3], V. James[3], C.W. Potter[1], K. Rogers[2] & G. Jacob[2]

*Departments of [1]Virology and [2]Surgery; The University of Sheffield Medical School; and [3]Blood Transfusion Service, Sheffield, UK.*

**Summary** In the present study we have confirmed that approximately one third of human colorectal carcinomas fail to express the HLA - A, B, C monomorphic determinant reactive with the W6/32 MAb, and 44% express class II HLA antigens as shown by reactivity with NFK-1 MAb. Reduced staining with the W6/32 MAb was not always associated with loss of $\beta$2m. In addition, the expression of HLA-A2 and Bw4 class I specific haplotypes on normal colon epithelium and tumour biopsy tissue was assessed. All normal colonic epithelia stained positively with MAb against A2 and Bw4 antigens, but a loss of these determinants was shown on tumour biopsies from patients tissue typed for the respective specificities. Loss of the A2 haplotype was shown in 4 of 15 tumour tissue samples, and loss of Bw4 specificities in 5 out of 7 tissue samples. The failure to detect specific loci determinants was not necessarily associated with loss of reactivity with W6/32 MAb.

Class I and class II MHC antigens are essential for the regulation of several immune functions. MHC class I antigens operate as restriction elements in T-cell mediated cytotoxicity, whilst class II antigens are involved in the presentation of antigen to cells of the immune system (Zinkernagel & Doherty, 1979; Benacerraf, 1981). Studies on the expression of HLA antigens on tumour cells using conventional tissue typing techniques have indicated that altered expression of HLA antigens can occur (Pollack *et al.*, 1980; 1981). More recently, monoclonal antibodies against HLA-A, B or C framework structures and the associated beta-2 microglobulin ($\beta$2m) have shown that a reduced expression, and in some cases a complete loss of class I antigens, is found in certain tumours (Fleming *et al.*, 1981; Daar & Fabre, 1983; Csiba *et al.*, 1984; Momberg *et al.*, 1986). Inappropriate expression of HLA class II antigens has been demonstrated in malignant melanoma, breast carcinoma and colorectal cancer (Natali *et al.*, 1983; Brocker *et al.*, 1984; Wilson *et al.*, 1984; Daar & Fabre, 1983; Rognum *et al.*, 1983).

In a recent study Van den Ingh *et al.* (1987) failed to demonstrate a correlation between the histological characteristics of colorectal tumours and either the abnormal expression of class II antigens or the loss of class I antigens; adenomas and non-mucinous carcinomas were shown to strongly express class I framework antigens. It is perhaps significant that mucinous tumours, which have a poor prognosis, showed loss of expression of HLA-antigens and low numbers of infiltrating lymphocytes.

An additional study by Momberg *et al.* (1986) has suggested that the loss of class I antigens from the surface of colorectal tumour cells is related to a lower degree of differentiation. These tumours, like the mucinous tumours, have a poor prognosis which may indicate that loss of class I determinants is associated with a more malignant phenotype. Although definitive evidence for this has not yet been documented, experiments in murine models of tumour growth and metastasis suggest that loss of class I expression may influence tumour progression. Transfection of class I genes into tumours lacking expression was found to hinder tumour growth and in some cases abrogate metastasis (Hui *et al.*, 1985; Wallich *et al.*, 1985). In the present study, we extend previous investigations by showing the loss of individual A and B locus HLA-A2 and Bw4 antigen specificities on colon carcinoma cells, which was not necessarily associated with a simultaneous loss of HLA monomorphic antigenic determinants. The HLA-A2 and Bw4 specific monoclonal antibodies were chosen because of the relatively high frequency of these antigens in the population, and their availability. The results presented do not indicate that these specificities are more prone to loss of expression than other HLA class I polymorphic determinants.

### Materials and methods

### *Patients and specimen collection*

Patients admitted to the study were undergoing laparotomy and resection of colorectal adenocarcinoma. Colorectal tumours were classified by Dukes' staging, as type A, B or C, depending on the degree of tumour invasion. Samples of tumour tissue and normal colon, taken from a site 15 cm distant from the tumour, were collected from each patient, wrapped in aluminium foil, sprayed with 'Supra-freezit' (Sorrisol, Merseyside) and snap-frozen. All tissue samples were stored at −80°C until frozen sections were cut using a cryostat at a thickness of 5–10 $\mu$m. A total of 30 patients were included in the study and the majority of these were tissue typed for HLA-A, B and C, using peripheral blood lymphocytes from the patient and conventional lymphocytotoxicity techniques (typing antisera and complement).

### *Immunoperoxidase staining of frozen sections*

Slides stored at −80°C were warmed to room temperature and rehydrated in Tris-buffered saline (TBS). Following the removal of excess TBS, 40 $\mu$l mouse monoclonal antibody (MAb) (at the appropriate dilution in TBS) was laid onto each section which was incubated for 40 min in a humidified atmosphere at room temperature. The sections were then washed twice in TBS prior to the addition of 50 $\mu$l rabbit anti-mouse peroxidase (Dako Ltd., Bucks.). The slides were covered and left for 40 min at room temperature and subsequently washed twice in TBS, flooded with diaminobenzidine (DAB) and with 0.5% (v/v) hydrogen peroxide for 10 min. Following washing of the slides in tap water they were stained in Harris's haematoxylin and mounted for examination under a light microscope.

### *Monoclonal antibodies*

The following monoclonal antibodies (MAbs) were used in the study: W6/32 directed against the monomorphic alpha chain determinant of HLA-A, B, and C framework antigens (Barnstable *et al.*, 1978), was a gift from Dr W.F. Bodmer, Imperial Cancer Research Fund, Lincoln's Inn Fields, London; anti-$\beta$2m was purchased from Sera-tech Limited: NFK-1 monoclonal antibody (Daar *et al.*, 1984), which

*Present address: Imperial Cancer Research Fund, Lincoln's Inn Fields, London WC2A 5PX, UK.
Correspondence: R.C. Rees.
Received 1 June; and in revised form, 22 October 1987.

recognises a monomorphic determinant of human class II antigens, including the products of the DR, DP and DQ loci, was a gift from Dr S. Fuggle, Nuffied Department of Surgery, John Radcliffe Hospital, Oxford: BB7.2 is an anti-HLA-A2 monoclonal antibody (Brodsky *et al.*, 1979) and was kindly donated by Dr Bodmer, Imperial Cancer Research Fund, London: 116.5.28 monoclonal antibody recognises the HLA-Bw4 specificity and this reagent was produced at Sheffield Blood Transfusion Centre by one of us (KG) (unpublished). Anti-cytokeratin monoclonal antibody was obtained from Amersham International plc, Bucks UK.

*Tissue typing*

HLA typing at A and B loci was performed using a standard microlymphocytotoxicity assay with reagents specific for the following antigens: A1, 2, 3, 9, 25, 26: 11, 28, 29, w30, w31: B5, 7, 8, 44, 45, 13, 14, 15, 16, 17, 18, 21, 22, 27, 35, 37 WHO.

### Results

*Expression of monomorphic and polymorphic HLA determinants on colon carcinoma cells*

Using the MAb W6/32, uniform staining of epithelial and interstitial cells in all normal colon mucosa was observed. Similar staining was seen with the anti-$\beta$2m MAb. In contrast, a proportion of the colon tumour specimens demonstrated variable staining patterns. In total, 7 out of 30 (23%) of the specimens examined showed loss of reactivity with MAb W6/32; one specimen showing a complete loss of reactivity with tumour cells, although in all cases the cells within the stroma remained strongly positive (data not shown). The remaining 23 tumour sections were uniformly positive with MAb W6/32. Only 2 of the 7 tumour samples which demonstrated heterogeneity of expression of the class I $\alpha$-chain also showed (in serial sections) a partial loss of reactivity with anti-$\beta$2m antibody. Using the MAb NFK-1, which reacts with a monomorphic determinant of the class II DP, DQ and DR antigens, none of the epithelial cells in normal colon samples obtained from the 30 patients entered into the study showed reactivity, although cells within the interstitium (probably macrophages and B-cells) were strongly stained. Forty-four per cent of the tumours examined demonstrated foci of positively stained cells with NFK-1 MAb 14, and these results are consistent with those previously published. The epithelial nature of the cells in both normal and tumour sections was confirmed by staining serial sections with anti-cytokeratin MAb (data not shown).

Twelve of the 30 patients were shown to be positive for HLA-A2 by conventional tissue typing techniques, and a further three, who were not tissue typed, were included because their normal colon epithelium stained positively with MAb BB7.2 (specific for HLA-A2). With all patients, normal colon epithelium showed intense staining with MAb W6/32 and MAb BB7.2 (Table I). Assessment of tumour sections from the patients revealed that 3 of the 15 specimens showed areas of positive and negative staining with BB7.2 antibody, and in one further sample all epithelial cells failed to react with this MAb. Two of the 4 tumour samples showing reduced expression of the HLA-A2 specificity also had areas of tumour cells which were negative with MAb W6/32, but the remaining two tumours showed an absence of the A locus specificity without detectable loss of the HLA class I framework antigen (W6/32 reactive, BB7.2 non-reactive).

Tissues from 7 patients, who were Bw4 positive by tissue typing were stained with the MAb 116.5.28 to determine

**Table I** Expression of HLA-A2 (BB7.2) and Bw4 (116.5.28) specificities on colorectal tumour and normal colon epithelium of patients positively typed for those respective antigens

| *Immunoperoxidase staining with monoclonal antibodies: Tissue:* | | | | | | |
|---|---|---|---|---|---|---|
| | | *Degree*[a] | *Normal colon epithelium* | | *Tumour* | |
| *Patient* | *Dukes' stage* | *of differentiation* | *W6/32* | *BB7.2* | *W6/32* | *BB7.2* |
| EB | B | W | + | + | +/− | +/− |
| RL | B | W | + | + | +/− | +/− |
| LN | B | M | + | + | + | − |
| IB | B | W | + | + | + | +/− |
| GB | B | M | + | + | + | + |
| CB | B | M | + | + | + | + |
| FB | C | W | + | + | + | + |
| FH | B | W | + | + | + | + |
| RP | C | M | + | + | + | + |
| CR | B | M | + | + | + | + |
| SS | B | M | + | + | + | + |
| RR | B | M | + | + | + | + |
| DT | C | W | + | + | + | + |
| EE | C | P | + | + | + | + |
| EJ | C | M | + | + | + | + |
| | | | W6/32 | 116-5-28 | W6/32 | 116-5-28 |
| HD | C | W | + | + | +/− | +/− |
| VP | B | M | + | + | − | − |
| FB | B | W | + | + | + | +/− |
| SS | B | M | + | + | + | +/− |
| AR | C | M | + | + | + | +/− |
| HS | A | W | + | + | + | + |
| CB | B | M | + | + | + | + |

+, Section uniformly stained with antibody.

−, Negative staining of epithelial cells; +/−, section demonstrating areas of positively and negatively stained cells.

[a]Tumours classified as well (W), moderately (M) or poorly (P) differentiated adenocarcinomas.

**Figure 1** Tumour tissue from a patient (LN) tissue typed as HLA-A2 positive; *Upper panel*: Adenocarcinoma cells stained uniformly with W6/32 MAb.; *Lower panel*: Loss of staining reaction with MAb BB 7.2 (anti-HLA-A2 specific), but stromal cells stain positively.

whether tumour tissue demonstrated an altered expression of the B locus of class I. Normal colon epithelium was stained positively with both W6/32 and 116.5.28 MAbs (Table I), whereas the tumour samples from these patients showed a variable staining pattern. Of the 7 tumours examined, 4 showed a partial loss of 116.5.28 reactivity and one a complete absence of detectable antigen. Three samples that showed negative staining with MAb 116.5.28, reacted in consecutive sections with MAb W6/32, indicating that although a monomorphic antigen sequence of HLA class I was present, the Bw4 specificity had been lost. Figure 1 shows an example of tumour stained uniformly with W6/32 MAb, but loss of reactivity with the BB7.2 MAb (anti-HLA-A2 specific). Of the tumours showing a loss of HLA-A2 specificity, 3 out of 4 were well differentiated whereas the majority of tumours showing no loss of antigenic expression were moderately differentiated (only 3 out of 11 were well differentiated). Within the group of 5 tumours showing a loss of HLA-Bw4 antigen specificity, 2 out of 5 were well differentiated (Table I). Tumours showing a loss of polymorphic HLA determinants were classified mostly as Dukes' stage B (7 out of 9 tumours were Dukes' B), although no definite correlation could be established between loss of specificity and disease stage.

### Discussion

Cytotoxicity by T lymphocytes towards neoplastic cells relies on the co-expression of specific antigenic determinants and class I histocompatibility antigens (Zinkernagel and Doherty, 1979). Class I and II MHC antigens also have a role in the induction and modulation of immune reactivity, including the presentation of foreign antigens to class II restricted helper T lymphocytes and target cell recognition by class I restricted cytotoxic T lymphocytes. It is therefore important to determine the aberrant expression of MHC antigens in disease states, and many studies have shown that significant changes in both class I and class II HLA antigens occur in malignant disease (Fleming *et al.*, 1981; Bhan and DesMarais, 1983; Whitwell *et al.*, 1984; Rowe & Beverley, 1984; Daar & Fabre, 1983; Csiba *et al.*, 1984; Umpleby *et al.*, 1985; Momberg *et al.*, 1986; Daar *et al.*, 1982).

Approximately one third of colorectal tumours show reduced staining with W6/32 MAb, which reacts with HLA-A, B, C framework determinants (Momberg *et al.*, 1986), and this finding shows a relationship with poorly differentiated tumours. Our results, showing a loss of W6/32 reactivity are consistent with previous reports.

The present study has also confirmed that class II HLA antigens are expressed in approximately one third of the tumours, and these results are similar to those reported by others (Daar *et al.*, 1982; Daar & Fabre, 1983; Csiba *et al.*, 1984). Recently the distribution of the D-region sub-locus products in colorectal cancer has been reported (Ghosh *et al.*, 1986), where DR appeared as the prominent specificity, but the expression of DR, DP and DQ determinants failed to correlate with disease stage or degree of differentiation.

In previous reports MAbs have been used which showed specificity for non-polymorphic determinants of HLA class I molecules, where any loss of individual specificities of the HLA A or B locus would not have been detected. In the present study we have used two MAbs with specificity for the HLA-A2 and HLA-Bw4 determinants (Brodsky *et al.*, 1979; Gelsthorpe, unpublished). Patients were tissue typed for A2 or Bw4 either by conventional tissue typing, or, as in three cases, by staining of normal colon mucosa with an A2 specific MAb. This allowed us to establish a group of 15 patients of known A2 haplotype, and 7 of Bw4 specificity. To our knowledge, the loss of individual specificities on colorectal tumours has not been previously reported. The results show that loss of these determinants can occur without the simultaneous loss of HLA framework determinants reactive with the W6/32 MAb, or with anti-$\beta$2m MAbs. Thus, of three tumour biopsies showing a partial, and one a complete loss of A2 specificity, two samples stained intensely with W6/32 MAb. In addition, 7 patients were tissue typed as Bw4 positive, and tumours from 4 of these showed a partial loss of reactivity with MAb 116.5.28 and one a complete loss of reactivity; however, in 3 of the tumours the absence of the specific Bw4 determinant was not associated with a loss of reactivity to MAb W6/32.

The loss of A or B locus determinants of HLA class I antigens is of considerable interest, particularly in relation to the possible interaction of these specificites with cytotoxic T lymphocytes, where absence of defined HLA-A or -B specificities could theoretically influence T-cell antigen recognition and cytotoxic capability. It is not clear whether the failure to express individual class I loci is a consequence of alteration in antigenic structure and spacial arrangement, a failure to transcribe genetic information coding for the subdomain region of the HLA molecule, or possibily the masking of epitopes expressed on finite specifities resulting in the failure of the monoclonal antibody to recognise and bind to that epitope. This latter explanation is less likely, since loss of antigen expression was associated with distinct 'foci' of epithelial cells which were completely unreactive with MAbs. From this and recent studies, it is evident that primary colorectal carcinoma cells are heterogeneous with regard to framework and A and B locus antigen expression, although it is not clear whether altered HLA antigen expression is associated with other biological properties such as tumour invasion and metastases. The finding that polymorphic regions of HLA class I antigens may be lost in tumours underlines the complex nature of HLA expression.

This work was supported by the Yorkshire Cancer Research Campaign. We are grateful to Mrs C. Mullan for typing the manuscript, and to the technical staff of the Tissue Typing Laboratory, Blood Transfusion Service, Sheffield, for tissue typing the patients admitted to this study.

### References

BARNSTABLE, C.J., BODMER, W.F., BROWN, G. & 4 others (1978). Production of monoclonal antibodies to group A erythrocytes. HLA and other human cell surface antigens-new tools for genetic analysis. *Cell*, **14**, 9.

BENACERRAF, B. (1981). Role of MHC gene products in immune regulation. *Science*, **212**, 1229.

BHAN, A.K. & DESMARAIS, C.L. (1983). Immunohistologic characterization of major histocompatibility antigens and inflammatory cellular infiltrate in human breast cancer. *J. Natl Cancer Inst.*, **71**, 507.

BROCKER, E.-B., SUTER, L. & SORG, C., (1984). HLA-DR antigen expression in primary melanomas of the skin. *J. Invest. Dermatol.*, **82**, 244.

CSIBA, A., WHITWELL, H.L. & MOORE, M. (1984). Distribution of histocompatibility and leucocyte differentiation antigens in normal human colon and in benign and malignant colonic neoplasms. *Br. J. Cancer*, **50**, 699.

DAAR, A.S., FUGGLE, S.V., TING, A & FABRE, J.W. (1982). Anomalous expression of HLA-DR antigens on human colorectal cancer cells. *J. Immunol.*, **129**, 447.

DAAR, A.S. & FABRE, J.W. (1983). The membrane antigens of human colorectal cancer cells: demonstration with monoclonal antibodies of heterogeneity within and between tumours and of anomalous expression of HLA-DR. *Europ. J. Cancer clin. Oncol.*, **19**, 209.

DAAR, A.S., FUGGLE, S.V., FABRE, J.W., TING, A. & MORRIS, P.J. (1984). The detailed distribution of MHC class II antigens in normal human organs. *Transplantation*, **38**, 293.

FLEMING, K.A., McMICHAEL, MORTON, J.A., WOODS, J. & McGEE, J.O'D. (1981). Distribution of HLA class I antigens in normal human tissue and in mammary cancer. *J. Clin. Pathol.*, **34**, 779.

GHOSH, A.K., MOORE, M., STREET, A.J., HOWAT, J.M.T. & SCHOFIELD, P.F. (1986). Expression of HLA-D sub-region products of human colorectal carcinoma. *Int. J. Cancer*, **38**, 459.

HUI, K., GROSVELD, F. & FESTENSTEIN, H. (1985). Rejection of transplantable AKR leukaemia cells following MHC DNA mediated cell transformation. *Nature*, **311**, 750.

MOMBERG, F., DEGENER, T., BACCHUS, E., MOLDENHAUSER, G., HAMMERLING, G.J. & MOLLER, P. (1986). Loss of HLA-A, B, C and *de novo* expression of HLA-D in colorectal cancer. *Int. J. Cancer*, **37**, 179.

NATALI, P.G., GIACOMONI, P., BIGOTTI, A. & 4 others (1983). Heterogeneity in the expression of HLA and tumour-associated antigens by surgically removed and cultured breast carcinoma cells. *Cancer Res.*, **43**, 660.

POLLACK, M.S., HEAGNEY, S. & FOGH, J. (1980). HLA typing of cultured human cell lines: The detection of genetically appropriate HLA-A, B, C and DR alloantigens. *Transplant. Proc.*, **12**, 134.

POLLACK, M.S., SOCORRO, D., HEAGNEY, D., LIVINGSTON, P.O. & FOGH, J. (1981). HLA-A, B, C and DR alloantigen expression on 46 cultured human cell lines. *J. Natl Cancer Inst.*, **66**, 1008.

ROGNUM, T.O., BRANDTZAEG, P. & THORUD, E. (1983). Is heterogeneous expression of HLA-DR antigens and CEA along with DNA-profile variations evidence of phenotypic instability and clonal proliferation in human large bowel carcinomas? *Br. J. Cancer*, **48**, 543.

ROWE, D.J. & BEVERLEY, P.C.L. (1984). Characterisation of breast cancer infiltration using monoclonal antibodies to human leucocyte antigens. *Br. J. Cancer*, **49**, 149.

UMPLEBY, H.C., HEINEMANN, D., SYMES, M.O. & WILLIAMSON, R.C.N. (1985). Expression of histocompatibility antigens and characterization of mononuclear cell infiltrates in normal and neoplastic colorectal tissues of humans. *J. Natl Cancer Inst.*, **74**, 1161.

VAN DEN INGH, H.F., RUITER, D.J., GRIFFIOEN, G., VAN MUIJEN, G.N.P. & FERRONE, S. (1987). HLA antigens in colorectal tumours – low expression of HLA class I antigens in mucinous colorectal carcinomas. *Br. J. Cancer*, **55**, 125.

WALLICH, R., BULBUC, N., HAMMERLING, G.J., KATZAV, S., SEGAL, S. & FELDMAN, M. (1985). Abrogation of metastatic properties of tumour cells by *de novo* expression of H-2K antigens following H-2 gene transfection. *Nature*, **315**, 301.

WHITWELL, H.L., HUGHES, H.P.A., MOORE, M. & AHMED, A. (1984). Expression of major histocompatibilty antigens and leucocyte infiltration in benign and malignant human breast disease. *Br. J. Cancer*, **49**, 161.

WILSON, B.S., HERZIG, M.A. & LLOYD, R.V. (1984). Immuno-peroxidase staining for la-like antigens in paraffin-embedded tissues from human melanomas and lung carcinoma. *Amer. J. Pathol.*, **115**, 102.

ZINKERNAGAL, R.M. & DOHERTY, P.C. (1979). MHC cytotoxic T-cells: Studies on the biological role of polymorphic major transplantation antigens determining T-cell restriction – specificity, function and responsiveness. *Adv. Immunol.*, **27**, 51.